# Phagocytic and pinocytic uptake of cholesterol in *Tetrahymena thermophila* impact differently on gene regulation for sterol homeostasis

**DOI:** 10.1038/s41598-021-88737-z

**Published:** 2021-04-27

**Authors:** Josefina Hernández, Matías Gabrielli, Joaquín Costa, Antonio D. Uttaro

**Affiliations:** Instituto de Biología Molecular y Celular de Rosario, CONICET, Facultad de Ciencias Bioquímicas y Farmacéuticas, Universidad Nacional de Rosario, S2000FHQ Ocampo y Esmeralda, Rosario Argentina

**Keywords:** Lipids, Cellular microbiology, Transcription

## Abstract

The ciliate *Tetrahymena thermophila* can either synthesize tetrahymanol or when available, assimilate and modify sterols from its diet. This metabolic shift is mainly driven by transcriptional regulation of genes for tetrahymanol synthesis (TS) and sterol bioconversion (SB). The mechanistic details of sterol uptake, intracellular trafficking and the associated gene expression changes are unknown. By following cholesterol incorporation over time in a conditional phagocytosis-deficient mutant, we found that although phagocytosis is the main sterol intake route, a secondary endocytic pathway exists. Different expression patterns for TS and SB genes were associated with these entry mechanisms. Squalene synthase was down-regulated by a massive cholesterol intake only attainable by phagocytosis**-**proficient cells, whereas C22-sterol desaturase required ten times less cholesterol and was up-regulated in both wild**-**type and mutant cells. These patterns are suggestive of at least two different signaling pathways. Sterol trafficking beyond phagosomes and esterification was impaired by the NPC1 inhibitor U18666A. NPC1 is a protein that mediates cholesterol export from late endosomes/lysosomes in mammalian cells. U18666A also produced a delay in the transcriptional response to cholesterol, suggesting that the regulatory signals are triggered between lysosomes and the endoplasmic reticulum. These findings could hint at partial conservation of sterol homeostasis between eukaryote lineages.

## Introduction

Sterols are important components of cell membranes in most eukaryotic lineages. They are involved in the modulation of membrane fluidity and permeability. By interacting with lipids and proteins, they participate in cell processes such as signal transduction, as part of lipid rafts, endosomal recycling transport, caveolae-dependent endocytosis and phagocytosis. Sterols also serve as precursors for the synthesis of steroid hormones, vitamin D and cholic acids^1–6^. Although sterols are generally regarded as a hallmark of eukaryotic cells, the requirement of such compounds varies greatly between lineages^7,8^. Interestingly, phylogenetically divergent species, including some ciliates, excavates, the fungus *Piromyces* sp. and the polychaete worm *Alvinella pompejana*, neither synthesize nor require imported sterols for growth. Instead, these organisms produce the surrogate tetrahymanol, a polycyclic triterpenoid similar to bacterial hopanoids^9,10^.

While the model ciliate species *Tetrahymena thermophila* has lost the genes for de novo synthesis of sterols, it has conserved those involved in mevalonate and squalene synthesis. *Tetrahymena* acquired the capacity to synthesize tetrahymanol in one step without the need for molecular oxygen by means of the squalene-tetrahymanol cyclase (THC) activity^11–14^. Despite this functional substitution, *Tetrahymena* also possesses enzymes that modify dietary-source sterols, transforming them into the tri-unsaturated molecule 7,22-*bis*dehydrocholesterol (BDHC)^15–19^. This metabolic flexibility makes cell survival possible in different environmental conditions: while tetrahymanol allows cells to adapt to poorly oxygenated waters, sterols are preferably incorporated and bioconverted in oxygen-rich and sterol abundance conditions^10^. In the case of the latter, increasing levels of sterols inside the cell provoke the interruption of tetrahymanol synthesis, leading to tetrahymanol eventually being replaced by BDHC in the plasma membrane, among other lipid rearrangements^20,21^.

Sterol conversion and its influence on tetrahymanol production has been studied since the 1960s, but only recently has the identification and characterization of the four enzymes involved in BDHC synthesis been achieved. Included in these findings are two desaturases that insert double bonds at the C5 and C7 positions of the sterols B ring (DES5 and DES7, respectively)^16,18^, a de-ethylase that removes the ethyl group from the aliphatic lateral chain of phytosterols (DES24)^17^ and two paralogs of a novel desaturase which introduce double bonds at the C22 position of the lateral chain (DES22A and B)^19^. Both DES22 genes were identified through the analysis of transcriptome data from *T. thermophila* cells grown in the absence and presence of cholesterol. This analysis further demonstrated that tetrahymanol synthesis was repressed at multiple steps via the down-regulation of genes of both the mevalonate and the squalene biosynthetic pathways, primarily squalene synthase (*SQS)* and *THC*. Interestingly, *DES5*, *DES7*, *DES22A* and *DES22B* were significantly up-regulated by cholesterol^19^, strongly suggesting that *T. thermophila* possesses a sterol-sensing system that drives these transcriptional responses. No evidence is available regarding sterol transcriptional modulation among ciliates, likely due to other representatives of the phylum lacking the enzymes for sterol bioconversion and/or do not synthesize tetrahymanol^9^.

There is scarce information on how dietary-source sterols are internalized in *T. thermophila*. It has been reported in recent work that sterol incorporation in the ciliate mainly occurs by phagocytosis^22^. In this single experiment, images obtained by fluorescence microscopy using a BODIPY-cholesterol probe showed that in comparison with a conditional mutant in phagocytosis (II8G-IA) at the restrictive temperature^22,23^, only a phagocytosis proficient strain (CU428) was able to accumulate sterols after a 16-h incubation period.

Mammalian cells acquire exogenous cholesterol from receptor-mediated endocytosis of LDL particles that contain cholesterol primarily as cholesteryl-esters. These esters are hydrolyzed in endosomes, producing free cholesterol, which is then attached to the luminal protein Niemann-Pick Type C Disease 2 (NPC2). In late endosomes/lysosomes (LE/LY), NPC2 transfers its cargo to the membrane protein NPC1^3^. NPC1 makes cholesterol available for transfer to other sterol**-**binding proteins outside the LE/LY that are involved in delivering cholesterol and its metabolites to different cellular compartments^24,25^. Parasitic protozoa such as apicomplexan alveolates (*Plasmodium* sp., *Toxoplasma gondii*) or excavates (*Trypanosoma* sp.) also scavenge cholesterol from their host by internalizing LDL particles, although there is scarce information on the mechanisms for its intracellular trafficking^26–28^. These mechanisms are likely to differ from those present in free-living protists where other sterol sources are available.

Considering there is still a gap in the understanding of sterol import, intracellular transport and signaling systems in different eukaryotic lineages, the present study provides evidence of two alternative endocytic pathways for cholesterol uptake in the free-living ciliate *T. thermophila* as well as their involvement in the triggering of two putative different signaling routes for transcriptional regulation.

## Results

### A secondary pathway for cholesterol transport in *T. thermophila*

**Figure 1:**
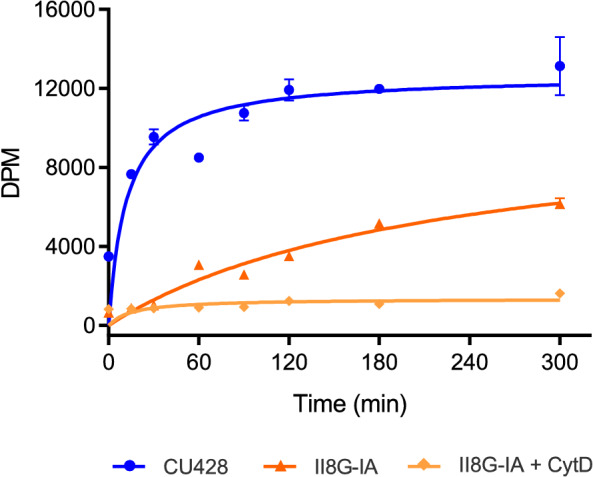
Kinetics of radiolabeled cholesterol incorporation in *T. thermophila* wild**-**type (CU428) and phagocytosis-deficient (II8G-IA) strains. Exponentially growing cells cultured at 37 °C were incubated with 26 μM, 1.54 mCi/mol [^14^C] − cholesterol. Samples of identical volume were collected at various time points and radioactivity was measured on cell pellets as disintegrations per minute (DPM). Cytochalasin D (CytD, 2 μM final concentration) was added 30 min before cholesterol. Data are expressed as mean and range of two replicates from a representative experiment, repeated three times with similar results.

Recent evidence suggests that cholesterol can primarily be internalized by the ciliate via phagocytosis^22^. In order to further characterize this process, we analyzed the kinetics of cholesterol intake. Radiolabeled cholesterol dissolved in ethanol was added to cultures at early exponential growth, and its incorporation into the cells was assessed at different times. Figure 1 shows that wild-type cells (CU428) avidly incorporated cholesterol with a hyperbolic behavior, reaching its maximum after approximately two hours. In contrast, the conditional mutant lacking phagosomes (II8G-IA)^23^ exhibited a reduced uptake of cholesterol at the restrictive temperature (37 °C). This uptake was completely abolished by cytochalasin D (Fig. 1) or latrunculin A (Supplementary Fig. S1), which are known inhibitors of actin assembly and actin-dependent vesicle transport^29,30^. This suggests the presence of a secondary pinocytic entry of sterols aside from the main phagocytic mechanism.

### Different signaling pathways triggered by cholesterol

**Figure 2:**
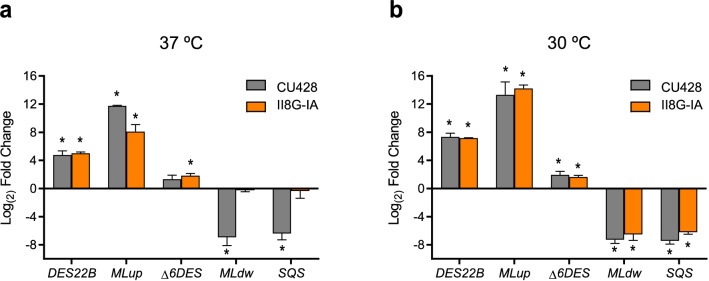
Effect of cholesterol on gene expression depends on the uptake route. Cells of wild-type (CU428) and phagocytosis-deficient (II8G-IA) strains were cultured at 37 °C (**a**) or 30 °C (**b**), and treated with 26 μM cholesterol 1 h prior to RNA extraction. Expression of representative genes involved in sterol and lipid metabolism which are differentially regulated was analyzed by RT-qPCR. Induced genes: *DES22B*, putative sterol C22 desaturase B; *MLup*, up-regulated ML domain protein; *Δ6DES*, delta-6 fatty acid desaturase. Repressed genes: *SQS*, squalene synthase; *MLdw*, down-regulated ML domain protein. Data are expressed as mean ± SD (n = 3) log_2_ fold change relative to untreated cells of the respective strain. *p < 0.05 vs. untreated cells, Student’s t test.

The next question we addressed was whether the sterol intake route would influence the transcriptional response of genes known to be regulated by the presence of cholesterol in *T. thermophila*. In our previous transcriptomic analysis^19^, we found that cholesterol produced the up-regulation of 179 genes and the down-regulation of 177 genes. Among these, we selected several up- and down-regulated genes to quantify their transcripts by RT-qPCR in cells grown in the absence or presence of cholesterol. Figure 2 shows the variation produced by cholesterol in transcript levels of *DES22B* (TTHERM_00085010), *MLup* (TTHERM_00030420) and *Δ6DES* (TTHERM_00339850) as representative up-regulated genes and *SQS* (TTHERM_00382150) and *MLdw* (TTHERM_00353379) as down-regulated genes^19^. RNA samples were obtained from CU428 and II8G-IA strains cultured at the restrictive (37 °C, Fig. 2a) or permissive (30 °C, Fig. 2b) temperatures. *MLdw* and *SQS* exhibited similar responses to cholesterol in both strains grown at 30 °C. Interestingly, these genes were only insensitive to cholesterol at the restrictive temperature in the II8G-IA strain, indicating that phagocytosis must be functional for a successful down-regulation of these genes. By contrast, up-regulated genes displayed similar responses to cholesterol in both strains and temperatures. The disparity in the transcriptional responses to cholesterol between these groups of genes further demonstrates that they are independently regulated by at least two signaling pathways.

**Figure 3:**
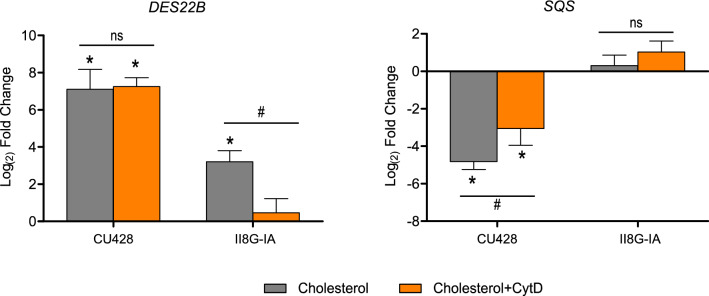
Effects of Cytochalasin D on the expression of cholesterol-regulated genes in wild**-**type (CU428) and phagocytosis-deficient (II8G-IA) strains. Cultures at exponential growth phase were incubated with 2 μM Cytochalasin D (CytD) or vehicle for 30 min prior to the addition of 26 μM cholesterol. Total RNA was extracted after 1 h of treatment with cholesterol and gene expression was analyzed by RT-qPCR. Data are expressed as mean ± SD (n = 3) log_2_ fold change relative to untreated cells of the respective strain. * p < 0.05 vs. untreated cells; # p < 0.05; ns, not significant difference; One-way ANOVA with Tukey post hoc test.

These results showed that up-regulated genes were induced in the II8G-IA strain despite the absence of phagosomal function. We considered the possibility that endocytosed cholesterol-loaded vesicles would arrive to an internal compartment where the signals would be triggered. Consequently, we examined the expression levels of up-regulated genes in both strains exposed to pharmacological treatment in order to disrupt any actin-dependent vesicular transport. *DES22B* and *SQS* were selected as representative reporters for up- and down-regulated genes, respectively, for further RT-qPCR determinations. As can be seen in Fig. 3, cytochalasin D abolished the response to cholesterol of *DES22B* in II8G-IA cells. The persistent induction of *DES22B* observed in CU428 cells could be due to an incomplete inhibition of endocytosis. As shown in Fig. 1 and Supplementary Fig. S1, the drugs completely prevented cholesterol internalization in mutant cells. These findings demonstrate that modulation of the transcriptional response to cholesterol requires its internalization by phagocytosis and pinocytosis, the latter being sufficient to induce *DES22B*. Furthermore, they exclude any involvement of signaling triggered from outside of the cell.

### Differential effect of cholesterol concentration in transcriptional regulation

**Figure 4:**
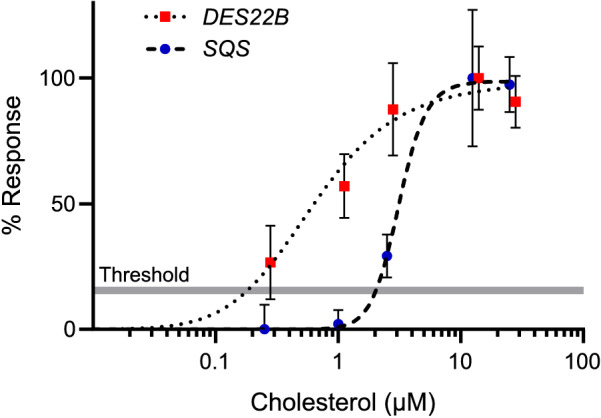
Cholesterol concentration-dependent response curves for the expression of *DES22B* and *SQS* in wild**-**type cells. Cultures were supplemented with different concentrations of cholesterol between 0.25 to 25 µM and RNA was extracted after 1 h for RT-qPCR analysis. Data represent the percentage of the maximum log_2_ fold change relative to untreated cells and are shown as mean ± SD (n = 3). Note that although *SQS* expression is repressed by cholesterol**,** the response here is plotted as positive values in order to allow a better visualization and comparison of the curves. The grey line indicates the threshold above which expression changes are considered significative.

The results of the previous section led to the hypothesis that only bulk incorporation of cholesterol can trigger the transcriptional repression of *SQS*. In contrast, *DES22B* up-regulation could be achieved with lower quantities of the sterol, which have been sufficiently provided by the pinocytic pathway. To support this idea we investigated the dependence of each signaling pathway on cholesterol concentration. Figure 4 shows that the cholesterol concentration required for a significant *SQS* down-regulation was approximately one order of magnitude higher than what was needed for *DES22B* up-regulation.

### Cell compartments involved in cholesterol signaling

We then investigated whether sterol intracellular distribution in *T. thermophila* resembles the ones known to date. We based our assumptions on the fact that its genome contains several putative orthologs of proteins involved in cholesterol homeostasis, i.e. an acyl-CoA:cholesterol acyl transferase (ACAT) and a protein (TTHERM_00672270) sharing 50% similarity to human NPC1^19^, suggesting the presence of a conserved mechanism for cholesterol delivery in the ciliate.

**Figure 5:**
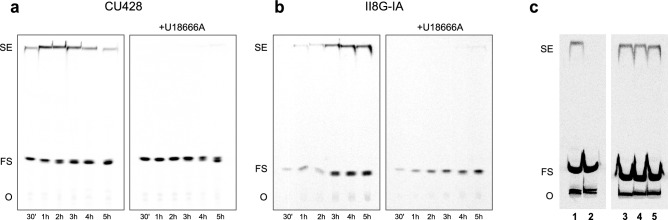
U18666A inhibits cholesterol esterification in *T. thermophila* without affecting ACAT. (**a**,**b**) Autoradiograms of TLC plates showing the sterol profile at different times after the addition of radiolabeled cholesterol in the presence or absence of U18666A in wild**-**type (**a**) and phagocytosis-deficient strains (**b**). Total lipids were extracted at various time points from cultures at exponential growth phase preincubated with 2 μM U18666A or vehicle for 15 min before the addition of 26 μM, 0.77 mCi/mol [^14^C] − cholesterol. (**c**) Autoradiograms show radiolabeled steryl esters synthesized by ACAT activity in vitro*.* Cell-free extracts were prepared from wild**-**type cells incubated overnight in the presence of 26 µM cholesterol as indicated in Methods section. ACAT activity was determined by the addition of 260 µM, 0.5 mCi/mmol [^14^C] − cholesterol and incubation for 1 h at 37 °C. The assays were performed in the absence (lanes **1** and **3**) or presence of 20 µM U18666A (lane **4**). In a parallel experiment, overnight cultures were treated for 20 min with 2 µM U18666A before preparation of extracts (lane **5**). A supernatant incubated for only 30 s (lane **2**) was used as negative control. Lipids were extracted and separated by TLC in silica gel plates. *FS* free sterols, *SE* steryl esters, *O* origin. Results shown are representative experiments repeated three times with similar results.

Analysis of the lipids extracted at different times after [^14^C]-cholesterol supplementation revealed the presence of free sterols and cholesteryl-esters in both CU428 and mutant cells. Synthesis of cholesteryl-esters indicates the arrival of cholesterol to the endoplasmic reticulum (ER), where ACAT is located in the ciliate^31^. In our radiolabeling experiments, we were able to estimate the time of trafficking to ER in approximately 30 min for wild-type cells and 120 min for mutant cells (Fig. 5a,b). The amino-steroid U18666A is a known inhibitor of intracellular cholesterol trafficking in mammalian cells^32^. It binds NPC1, thus blocking the trafficking between LE/LY and other organelles like the ER^33^. Treatment with U18666A inhibited the synthesis of cholesteryl-esters in both strains, indicating a blockage in the traffic between LE/LY (or similar organelles) and the ER. This effect persisted during the five hours of the assay (Fig. 5). Based on these results, it can be inferred that the two routes of cholesterol uptake share a common NPC1-containing compartment before reaching the ER.

To rule out a direct inhibition of ACAT by U18666A as a possible cause for these results, we assayed its activity in cell-free extracts prepared in two different ways: (i) cell-free preparations incubated with this inhibitor during the enzyme assay; (ii) preparations from cells treated with U18666A during 20 min before extract preparation. No significant inhibition was detected in any case (Fig. 5c).

**Figure 6:**
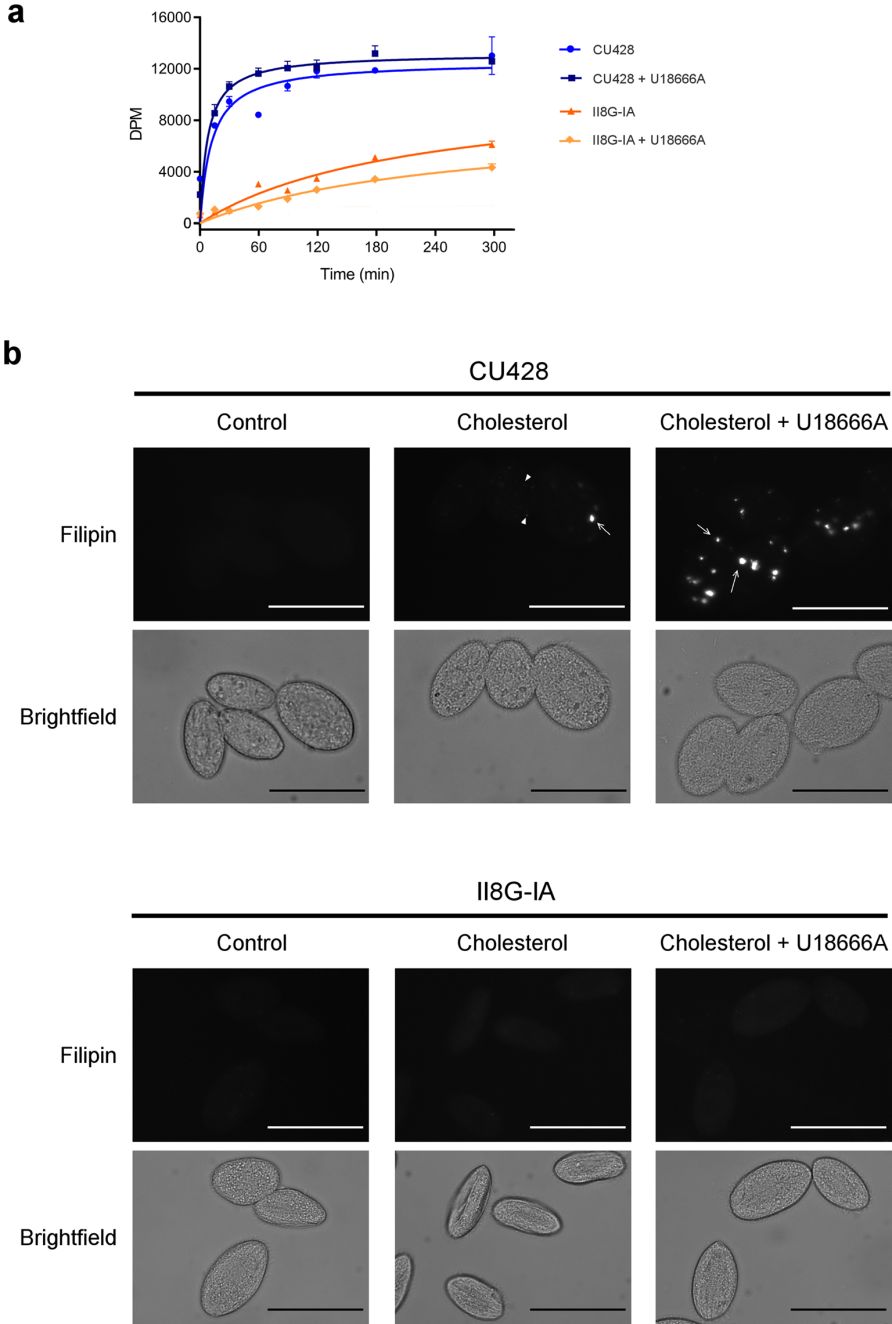
U18666A induces cholesterol accumulation in phagosome-like vesicles. (**a**) Kinetics of radiolabeled cholesterol incorporation in *T. thermophila* wild**-**type (CU428) and phagocytosis-deficient (II8G-IA) strains. The experiment was performed as described in legend of Fig. 1. U18666A (2 μM) was added 15 min before cholesterol. (**b**) Fluorescent microscopy. CU428 and II8G-IA cells were pretreated with 2 μM U18666A or vehicle for 15 min prior to the addition of 26 μM cholesterol. Untreated cells are designated as Control. After 1 h, cells were fixed, stained with Filipin and examined by fluorescence microscopy. Arrows indicate large, phagosome-like vesicles and arrowheads indicate selected small vesicles that were also observed in some cells. To improve visualization**,** some of the background fluorescence of CU428 cells was allowed, although II8G-IA cells required more contrast stretching as fluorescence levels were identical between treatments. Scale bar = 50 μm. Results shown are representative experiment repeated three times with similar results.

In agreement with previous reports that U18666A induced a transient accumulation of free cholesterol in mammalian lysosomes^3,33^, we observed in the present study a similar effect on *T. thermophila* (Fig. 6). The amount of radioactivity in the cell pellets increased shortly after treatment with U18666A in CU428 cells but not in mutant II8G-IA cells (Fig. 6a). To better understand the nature of this transient blockage, cells were stained with Filipin, a fluorescent dye that binds free, non-esterified sterols, and were observed using fluorescence microscopy. Wild-type cells treated with cholesterol for 1 h before fixation exhibited few characteristically large, phagosome-like vesicles (Fig. 6b, upper central panel). Pretreatment with U18666A produced a marked increase in the number and fluorescence intensity of such large vesicles (Fig. 6b, upper right panel). This supports the hypothesis that U18666A inhibits cholesterol transport from phagosomes or LE/LY to other vesicles or organelles, such as the ER.

In contrast with what was observed in wild**-**type cells, we could not detect Filipin staining signals in II8G-IA cells, as cell fluorescence in either treatment was similar to autofluorescence levels (Fig. 6b, lower panels). This lack in detection likely reflects the limitation of the method to detect small amounts of free cholesterol putatively present inside pinocytes or inserted in the plasma membrane. Under normal growth conditions, it is likely that cholesterol is rapidly distributed to different organelles and membranes, including the ER where it is esterified (and stored in lipid droplets). The fluorescent signal of Filipin therefore becomes fainter and more difficult to detect.

**Figure 7:**
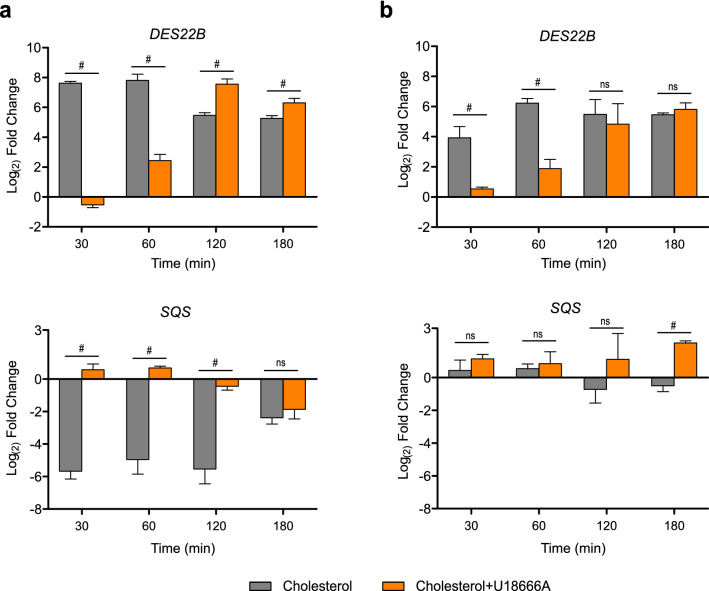
U18666A delays changes in the expression of cholesterol-regulated genes. Time course of the expression of *DES22B* and *SQS* in wild**-**type (**a**) or phagocytosis-deficient (**b**) cells. Cultures at exponential growth phase were incubated with 2 μM U18666A or vehicle for 15 min prior to the addition of 26 μM cholesterol. Total RNA was extracted at different times after treatment with cholesterol and gene expression was analyzed by RT-qPCR. Data are expressed as mean ± SD (n = 3) log_2_ fold change relative to untreated cells (before addition of U18666A or cholesterol) of the respective strain. Statistical analysis was performed by repeated measures two-way ANOVA with Bonferroni post hoc test to compare treatments at each time; # p < 0.05; ns, not significant difference.

We then tested whether the effects of U18666A are associated with changes in the transcriptional response to cholesterol. In the absence of the inhibitor, both *DES22B* and *SQS* genes showed a maximal response in CU428 cells 30 min after the addition of cholesterol. Treatment with U18666A produced a 60-min delay in the response of *DES22B* and completely reverted SQS down-regulation (Fig. 7a), similar to what was found in the untreated mutant (Fig. 7b). *DES22B* induction was also delayed in the mutant strain (30 min) and the NPC1 inhibitor produced a further delay in its up-regulation (Fig. 7b). These results are compatible with the localization of the signaling origin between LE/LY and ER for both up- and down-regulation. Alternatively, such signaling could be mediated directly by NPC1 in LE/LY.

### Effect of Brefeldin A on the transcriptional responses for both reporter genes

**Figure 8:**
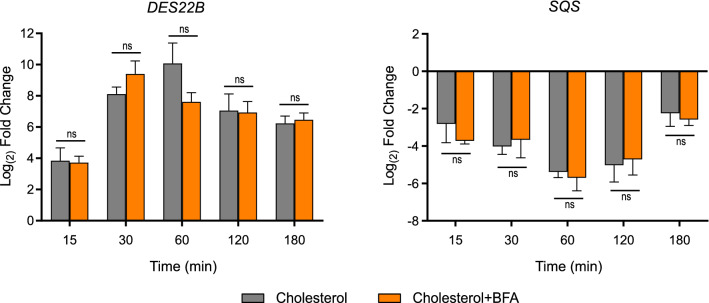
Effect of Brefeldin A on the expression of cholesterol-regulated genes. Time course of the expression of *DES22B* and *SQS* in wild**-**type cells. Cultures at exponential growth phase were incubated with 36 μM Brefeldin A (BFA) or vehicle for 2 h prior to the addition of 26 μM cholesterol. Total RNA was extracted at different times after treatment with cholesterol and gene expression was analyzed by RT-qPCR. Data are expressed as mean ± SD (n = 3) log_2_ fold change relative to cells before the addition of cholesterol. Statistical analysis was performed by repeated measures two-way ANOVA with Bonferroni post hoc test to compare treatments at each time; ns, not significant difference.

In order to support the notion that the triggering of signals is confined to the ER or in NPC1-containing compartments, we examined the effect of Brefeldin A, which has been used in *Tetrahymena* as a Golgi apparatus-disrupting agent^34^. We speculated that this agent would block cholesterol trafficking beyond the ER, thus increasing the cholesterol content in this cellular compartment and exacerbating the transcriptional response of reporter genes. Unexpectedly, Brefeldin A did not produce a significant increase in *SQS* down-regulation, although *DES22B* up-regulation was slightly increased after 30 min of cholesterol addition (Fig. 8). This response was quickly dissipated, as transcript levels of the gene were similar either with or without Brefeldin A at later time points. We were also unable to detect a significant increase in cholesterol conversion to steryl-esters (not shown). This result demonstrates that the Golgi apparatus is not involved in these particular signaling events.

## Discussion

*T. thermophila* sterol metabolism alternates between tetrahymanol synthesis and exogenous sterols uptake and bioconversion, likely as an adaptive mechanism to the changing content of oxygen in the aquatic medium and sterol abundance^9,10^. To produce this “switch”, the cell must rely on an internal signaling system that quickly and adequately responds to sterol availability.

In the present work, we demonstrate that cholesterol enters the cells through two types of actin-dependent transport: phagocytosis and pinocytosis. Our results support that phagocytosis is the main mechanism for cholesterol uptake, a finding which had also been suggested in a recent report^22^. Similar observations were made in the related ciliate *Paramecium primaurelia,* in which the uptake of a fluorescent cholesteryl ester analogue occurred via phagocytosis and through the plasma membrane^35^. Although we could not detect cholesterol incorporation in phagocytosis-deficient cells with the fluorescence microscopy approach used in these reports, we did so by measuring the uptake of radiolabeled cholesterol, which was found to be a more sensitive method. Because of their complex cortical organization, pinocytosis in *Tetrahymena* and other ciliates takes place at particular cellular sites, such as the parasomal sacs located near the ciliary basal bodies^36^. The elucidation of the mechanisms involved in sterol uptake warrants further research.

Based on the evidence shown in cholesterol esterification and U18666A treatment experiments, we propose that most of the cholesterol internalized by both mechanisms converges on mature phagolysosomes before being delivered to the ER where it is bioconverted or esterified. Interestingly, the fusion between endocytic vesicles formed at the parasomal sacs and phagosomes has been described in *Paramecium*^37^. As suggested from the experiments with U18666A, a NPC1-like protein would be present in this compartment and involved in sterol transport. In mammalian cells, NPC1 is located in LE/LY, where it is thought to control cholesterol export to other organelles together with NPC2; inhibitors and mutations in these proteins lead to accumulation of sterols and other lipids in endosomes/lysosomes^3^. We observed a remarkably similar effect in *Tetrahymena* cells stained with Filipin after treatment with U18666A. In fact, a putative NPC1-like protein exists in the *T. thermophila* genome, and a proteomic study located it in phagososmes^38^. NPC1-like proteins were found in the apicomplexan parasites *Toxoplasma gondii* and *Plasmodium falciparum*, where they play important roles in pathogenicity^39,40^. Interestingly, the *Plasmodium* ortholog is not located in endosomes/lysosomes but in the plasma membrane^40^. We are currently carrying out different experimental approaches in order to confirm its identity, localization and role in *Tetrahymena* sterol trafficking.

In a previous study, we showed that cholesterol provokes a large-scale regulation of gene expression on *T. thermophila*^19^. Here, we demonstrated that this response depends mainly on intracellular sterol concentration, which correlates with the type of transport in the cell. In other words, phagocytic entry is the primary way through which large quantities of sterols can enter the cell and trigger the repression of tetrahymanol biosynthesis. This process was reflected in the transcriptional down-regulation of SQS—a key enzyme in the tetrahymanol biosynthetic pathway—, which only took place at higher cholesterol concentrations in the medium. Conversely, *DES22* and other up-regulated genes required ten times less cholesterol, allowing a rapid enzymatic conversion of exogenous sterols to BDHC. This indicates that these processes involve at least two signaling pathways, each regulating a different set of genes. In principle, this double sterol signaling pathway could ensure that the sterol requirements of cell membranes would be met, and in so doing define when tetrahymanol could be completely replaced by BDHC.

To date, it is unknown how *Tetrahymena* detects the presence of sterols and which proteins and mechanisms participate in the modulation of the transcriptional response triggered by these compounds. Genome analysis of *T. thermophila* shows no evidence of proteins homologous to those that constitute the SREBP/SCAP pathway (sterol regulatory element-binding protein/SREBP cleavage-activating protein), which in mammalian cells acts as a sterol sensor and regulates the expression of key enzymes required for cholesterol homeostasis^3,41^. In contrast, several putative members of other sterol**-**binding protein families have been identified in the *Tetrahymena* genome, such as START domain-containing proteins and ORPs. These proteins have been implicated in nonvesicular transport of sterols and in modulation of signaling processes^24,25^, but their role in ciliate cellular physiology remains to be elucidated. Despite these uncertainties, our results dismiss the participation of extracellular signaling, as inhibition of both phagocytosis and pinocytosis impaired the transcriptional response. The experiments with U18666A as well as the lack of effect of Brefeldin A further suggest that the internal signaling pathways originate between LE/LY and the ER. Alternatively, signal(s) could be triggered at the plasma membrane: (i) after the arrival of cholesterol delivered directly from LE/LY or (ii) after the arrival of modified cholesterol (BDHC) from the ER, without intervention of the Golgi apparatus.

Overall, the present work highlights some similarities in cholesterol transport and distribution in distant-related organisms such as ciliates and metazoans. Further study on the way in which transport events take place in *T*. *thermophila,* a simple but powerful organism in terms of experimental malleability, would aid in the understanding of the mechanisms involved in cholesterol transport and homeostasis in higher eukaryotes. In addition, this ciliate lacks any endogenous synthesis of sterols, which in other models can mask the interpretation of sterol traffic/signaling experiments.

## Materials and methods

### Cell culture and drug treatment

*T. thermophila* strains CU428 (mpr1-1/mpr1-1, VII) and II8G-IA (presumed phg − /phg −)^23^ were grown in 250 mL flasks containing 100 mL SPP medium composed of 1% w/v beef peptone, 0.1% w/v yeast extract, 0.2% w/v glucose and either 0.003% (for CU428) or 0.01% w/v ferric citrate (for II8G-IA). Cultures were carried out in a rotary shaker at 180–200 rpm and 30 °C (permissive temperature for II8G-IA) or 37 °C (restrictive temperature for II8G-IA), as indicated in the text. To confirm the II8G-IA phenotype, phagosome formation was assessed by incubating the cells with Chinese Ink for 15 min before microscopic examination. Once the cultures reached the early exponential phase of growth (3–5 × 10^5^ cells/mL), they were divided into 5 mL aliquots in capped tubes and treated with either cholesterol, pharmacological inhibitors or both. Cells were counted using a hemocytometer. Cholesterol (Sigma) was added to a final concentration of 26 μM from a 26 mM stock solution prepared in absolute ethanol. Cytochalasin D (Sigma) and U18666A (Cayman Chemicals) were added to a final concentration of 2 μM from 2 mM stock solutions in DMSO 15 − 30 min before the addition of cholesterol. Latrunculin A (Sigma) was added to a final concentration of 20 μM from a 20 mM stock 5 min before cholesterol^30^. Brefeldin A (Sigma) was administered to a final concentration of 36 μM, from a 36 mM stock solution, 2 h prior to cholesterol treatment^34^. Treatments with identical volumes of ethanol or DMSO were used as controls and solvents were kept under 1% v/v. Each treatment was carried out in three biological replicates.

### RNA purification

For each biological replicate, 3 × 10^5^ cells were collected by centrifugation at 2000× *g* for 2 min at 4 °C and the cell pellets were immediately resuspended in 0.5 mL of TriPure reagent (Roche). Total RNA was extracted as indicated by the manufacturer’s instructions and conserved at − 70 °C until use. RNA integrity was verified using gel electrophoresis in 1.5% agarose. Total RNA concentration was determined using a NanoVue spectrophotometer (GE Life Sciences).

### RT-qPCR

Reverse transcription was performed in a 20-µL reaction volume with 2 µg of total RNA, which was previously treated with RQ1 RNase-Free DNase (Promega), Oligo(dT)_18_ Primer (Invitrogen) and M-MLV Reverse Transcriptase (Invitrogen), according to the manufacturer’s instructions. Real-time PCR amplification was carried out in a Mastercycler Realplex thermal cycler (Eppendorf) in a 20-µL final reaction volume containing 10 ng of template cDNA, 0.5 µM of gene-specific primers (Supplementary Table S1) and 13 µL of commercial mix for qPCR (Biodynamics), which contains Taq DNA polymerase, dNTPs, Mg^2+^ buffer and green fluorophore. Two technical replicates were run for each biological replicate. Cycling conditions were 95 °C for 2 min, followed by 40 cycles of 95 °C for 15 s, 60 °C for 20 s and 72 °C for 20 s (measuring step). A melting curve was added as a final step in order to ensure specificity of the amplification. Gene expression levels were normalized to the abundance of 17S rRNA gene as a reference, and relative quantification was performed following the modified ∆∆Ct method^42^.

### Radiolabeled Cholesterol intake

A mixture of radiolabeled [4–^14^C] − cholesterol (PerkinElmer) and cholesterol to a final concentration of 26 μM and 1.54 mCi/mmol specific activity was added to the cell cultures after treatment with the different drugs or vehicle. Duplicate samples of 0.2 mL were taken at different times; cells were harvested by centrifugation at 2000 × *g* and washed in 1 mL of SPP medium containing 26 μM cholesterol. Cell pellets were then resuspended in 1 mL scintillation count medium Optiphase HiSafe3 (PerkinElmer). Total radioactivity was measured using a Wallac 1209 Rackbeta scintillation counter.

### TLC and count of lipid fractions

Neutral lipids were extracted according to the Bligh and Dyer method^43^ and resuspended in chloroform. Each extract was seeded in 1 cm lanes on a 13 × 15 cm silica-aluminum plate (Merck). The mobile phase consisted of a mixture of acetic acid:diethyl ether:hexane (1:20:80 v/v/v). The plate was left to air dry and was exposed on a photosensitive screen overnight (Imaging Plate BAS-MS 2025, 20 × 25 cm, Fujifilm). Autoradiography scanning was performed on Typhoon FLA 7000 (GE Life Biosciences). For better visualization, minor adjustments in brightness and contrast were performed on the entire image using the Fiji software (ImageJ)^44^. For radioactivity measurement, each spot was first visualized with a sublimated iodine spray, then scraped and directly resuspended in scintillation liquid. Full-length versions of the autoradiograms are included in Supplementary Fig. S2 and S3.

### ACAT Activity in cell-free extracts

Measurement of ACAT activity in cell-free extracts was performed using a modification of the method of Billheimer et al*.*^31^. To induce ACAT activity, CU428 cultures starting at 2 × 10^4^ cells/mL were grown at 30 °C for 22 − 24 h and incubated overnight with 26 µM cholesterol. Volumes containing ~ 5.4 × 10^5^ cells were centrifuged and each pellet resuspended in 300 µL of 0.1 M potassium phosphate buffer pH 7.4 containing 1 mM glutathione and protease inhibitors (Roche). Cells were lyzed using a Diagenode Bioruptor UCD-200 sonicator bath (9 cycles 30 s ON + 30 s OFF, power High), and cell debris and unbroken cells were removed by centrifugation at 5000 × *g* for 5 min at 4 °C.

ACAT activity was determined in 300 µL of cell-free supernatants supplemented with 260 µM [^14^C] − cholesterol (0.5 mCi/mmol) in the presence of either 20 µM U18666A or 0.1% DMSO and incubated for 1 h at 30 °C. The reaction was stopped via the addition of 3 mL of chloroform–methanol 2:1 (v/v), the lipids were extracted and steryl esters were separated from free sterols in TLC silica plates as indicated above. As a negative control, lipids were extracted from supernatants which had undergone insignificant incubation (less than one min). Alternatively, cultures were treated with 2 µM U18666A for 20 min before preparation of cell**-**free extracts. Full-length versions of the autoradiograms are included in Supplementary Information.

### Filipin staining and fluorescence microscopy

Filipin III (Santa Cruz Biotechnology) was dissolved in DMSO at 5 mg/mL, aliquoted and stored under N_2_ at − 80 °C until use. CU428 and II8G-IA cultures at ~ 3 × 10^5^ cells/mL were treated with cholesterol in the presence or absence of U18666A as indicated above. After 1 h of incubation, samples of 200 µL were transferred to microcentrifuge tubes, centrifuged at 1000 × *g*, washed with 1 mL of 10 mM Tris–HCl pH 7.5 and resuspended in 100 µL Tris–HCl. Cells were fixed through the addition of an equal volume of 4% paraformaldehyde—3.4% sucrose in phosphate buffer saline (PBS) for 15 min at room temperature. They were then washed twice with 400 µL PBS and resuspended in 100 µL PBS. Staining was performed with 50 µg/mL Filipin for 1 h at room temperature with rotation in a HulaMixer (Invitrogen). Finally, the cells were washed twice with 400 µL PBS and resuspended in 40 µL PBS.

Cells mounted under coverslips were observed and photographed using a Nikon Eclipse E-800 epifluorescence microscope equipped with an Andor Clara DR-1306 monochrome camera. Filipin staining was visualized using a UV-2A filter cube. After being taken, the images were processed and analyzed using the Fiji software^44^.

## Supplementary Information

Supplementary Information
