# Supplementary material for: Phagocytic and pinocytic uptake of cholesterol in Tetrahymena thermophila impact differently on gene regulation for sterol homeostasis
Source: Sci Rep. 2021 Apr 27;11:9067. doi: 10.1038/s41598-021-88737-z (PMC8079401; doi:10.1038/s41598-021-88737-z)
Supplement: Supplementary file 1 — Supplementary Information [file 41598_2021_88737_MOESM1_ESM.pdf]

SUPPLEMENTARY INFORMATION

Phagocytic and pinocytic uptake of cholesterol in *Tetrahymena thermophila* impact differently on gene regulation for sterol homeostasis

Josefina Hernández, Matías Gabrielli, Joaquín Costa and Antonio D. Uttaro

Supplementary Table S1. Primers used for RT-qPCR

Target ID	Gene Name		Sequence (5’-3’)
TTHERM_00085010	*DES22B*	Fwd	CCCTGGTGCTCATTGGACAA
		Rev	TGCTTGAATGCAACAAAGCCA
TTHERM_00382150	*SQS*	Fwd	TACTCACCTGCTTCCATCC
		Rev	AACCAACTGCTCCTGCTAC
TTHERM_00339850	*Δ6DES*	Fwd	ACCAAACCGAACACCACTTCT
		Rev	CCAGATGGGGCTATACTTGATGA
TTHERM_00030420	*MLup*	Fwd	CTTCCATGCACTGGCACATA
		Rev	TCCACCTCTTGTACTCAAGTTGA
TTHERM_00353379	*MLdw*	Fwd	TGCAGTACCTGAGAAAAGTGTCA
		Rev	TCACCAGGATCTGCTTCTTGAG
M10932	17S rRNA	Fwd	GAATTGACGGAACAGCACACC
		Rev	TCACTCCACCAACTAAGAACGGC

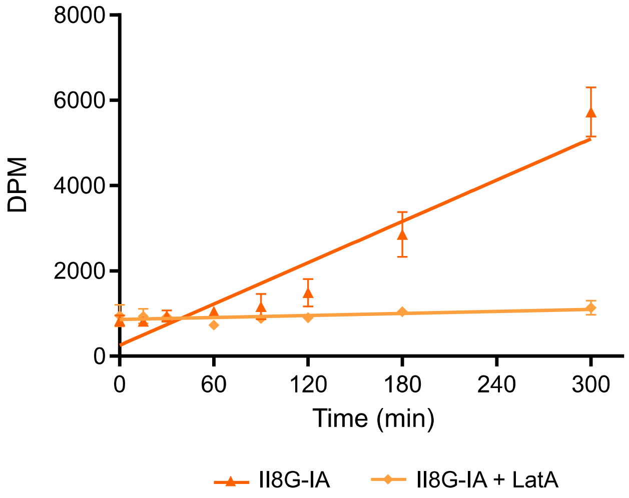

Supplementary Figure S1. Effect of latrunculin A on radiolabeled cholesterol incorporation in *T. thermophila* phagocytosis-deficient (II8G-IA) cells. Exponentially growing cells cultured at 37 °C were incubated with 26 µM, 1.54 mCi/mol [^{14}C]−cholesterol. Samples of identical volume were collected at various time points and radioactivity was measured on cell pellets. Latrunculin A (LatA, 20 µM final concentration) was added 5 min before cholesterol. Each data point represents the mean and range of two replicates.

a

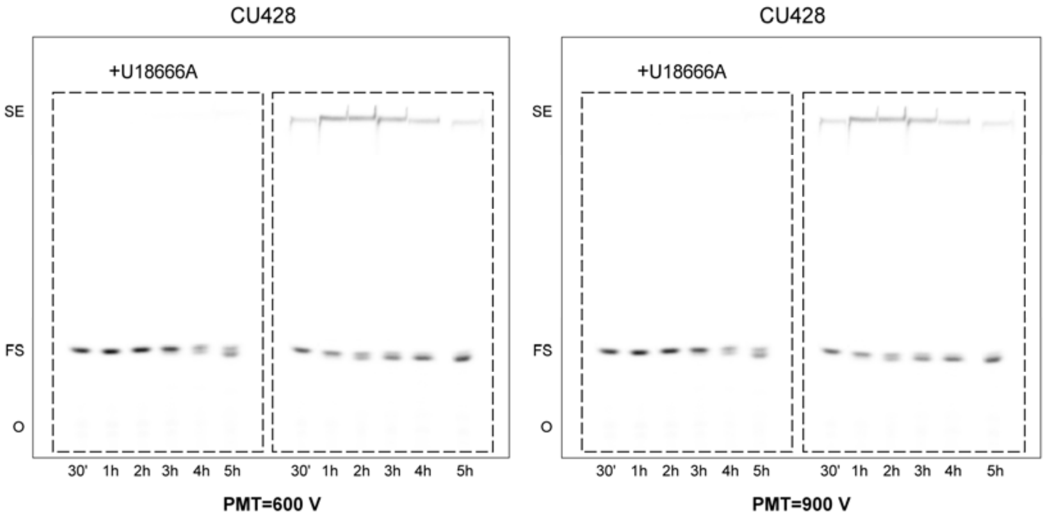
CU428
CU428
+U18666A
+U18666A
SE
FS
O
SE
FS
O
30' 1h 2h 3h 4h 5h 30' 1h 2h 3h 4h 5h
30' 1h 2h 3h 4h 5h 30' 1h 2h 3h 4h 5h
PMT=600 V
PMT=900 V

b

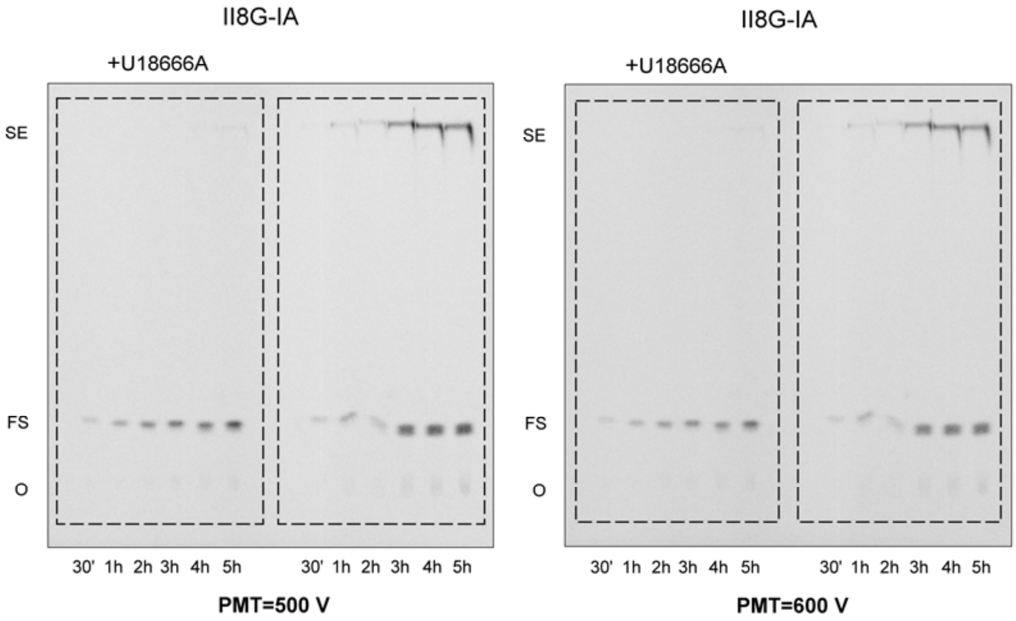
II8G-IA
II8G-IA
+U18666A
+U18666A
SE
FS
O
SE
FS
O
30' 1h 2h 3h 4h 5h 30' 1h 2h 3h 4h 5h
30' 1h 2h 3h 4h 5h 30' 1h 2h 3h 4h 5h
PMT=500 V
PMT=600 V

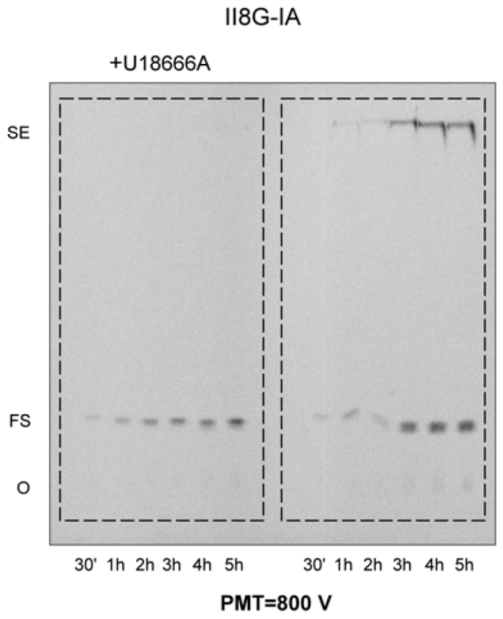

Supplementary Figure S2. Full-length autoradiograms corresponding to TLC plates shown in Figures 5a and 5b, respectively. Plates were digitized with a Typhoon FLA 7000 (GE Life Biosciences) scanner at 16-bit depth using different photomultiplier (PMT) settings. The continuous lines mark the outline of the each autoradiogram. The areas delimited by the dashed rectangles are shown separated in Figure 5 and in the opposite order (i.e. in each sub-figure, the area identified as "+U18666A" is presented on the right). Figure 5a shows the plate digitized at PMT=900 V from **a**, and in order to enhance visualization image maximum intensity was set to 19016 using ImageJ software Brightness/Contrast tool. Figure 5b shows the plate digitized at PMT=500 V from **b**, image minimum intensity was set to 7136 and maximum to 38040. SE, steryl esters; FS, free sterols O, origin.

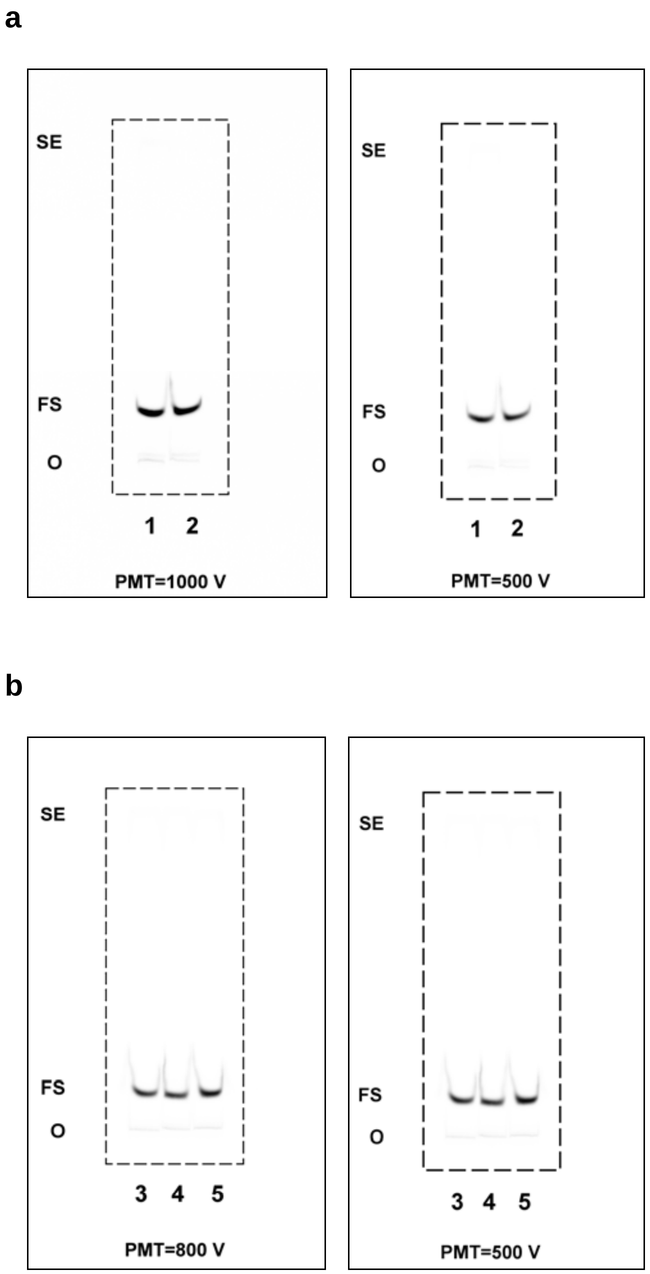

Supplementary Figure S3. Full-length autoradiograms of TLC plates shown in Figure 5c. Plates in **a** and **b** were digitized with a Typhoon FLA 7000 (GE Life Biosciences) scanner at 16-bit depth using different photomultiplier (PMT) settings. The areas delimited by the dashed rectangles of plates digitized at PMT=500 V are shown in Figure 5c and, in order to allow visualization of the fainter bands corresponding to cholesteryl esters (SE), images maximum intensity was set to 567 using ImageJ software Brightness/Contrast tool. Lane labels are the same as described in the Figure 5c legend. SE, steryl esters; FS, free sterols O, origin.
